# Patchiness of Ciliate Communities Sampled at Varying Spatial Scales along the New England Shelf

**DOI:** 10.1371/journal.pone.0167659

**Published:** 2016-12-09

**Authors:** Jean-David Grattepanche, George B. McManus, Laura A. Katz

**Affiliations:** 1 Department of Biological Sciences, Smith College, Northampton, Massachusetts, United States of America; 2 Department of Marine Sciences, University of Connecticut, Groton, Connecticut, United States of America; 3 Program in Organismic and Evolutionary Biology, University of Massachusetts, Amherst, Massachusetts, United States of America; Universidade de Aveiro, PORTUGAL

## Abstract

Although protists (microbial eukaryotes) provide an important link between bacteria and Metazoa in food webs, we do not yet have a clear understanding of the spatial scales on which protist diversity varies. Here, we use a combination of DNA fingerprinting (denaturant gradient gel electrophoresis or DGGE) and high-throughput sequencing (HTS) to assess the ciliate community in the class Spirotrichea at varying scales of 1–3 km sampled in three locations separated by at least 25 km—offshore, midshelf and inshore—along the New England shelf. Analyses of both abundant community (DGGE) and the total community (HTS) members reveal that: 1) ciliate communities are patchily distributed inshore (i.e. the middle station of a transect is distinct from its two neighboring stations), whereas communities are more homogeneous among samples within the midshelf and offshore stations; 2) a ciliate closely related to *Pelagostrobilidium paraepacrum* ‘blooms’ inshore and; 3) environmental factors may differentially impact the distributions of individual ciliates (i.e. OTUs) rather than the community as a whole as OTUs tend to show distinct biogeographies (e.g. some OTUs are restricted to the offshore locations, some to the surface, etc.). Together, these data show the complexity underlying the spatial distributions of marine protists, and suggest that biogeography may be a property of ciliate species rather than communities.

## Introduction

Microbial eukaryotes, or protists, play a critical role in plankton food webs by linking picoplankton (bacteria, archaea and very small eukaryotes) and Metazoa (copepods and fish) [1–3]). Recent studies have analyzed broad patterns of protist diversity in the global ocean using high-throughput sequencing (HTS), and observed that pico- and nano-sized organisms (0.2–20 μm in diameter) represent a large fraction of total plankton diversity, particularly within the rare biosphere [4–6]. This explains some of our gaps in knowledge about planktonic diversity, given that morphological identification of these small taxa is difficult compared to that of larger species. Given that DNA-based assessments of plankton diversity are possible, the tools are available to measure the variation in diversity on fine spatial scales, allowing us to link spatial heterogeneity (patchiness) to changes in food web structure, a critical characteristic of all ocean food webs.

We focus on ciliates, one of the most diverse protist clades in marine systems [4, 7]. In particular, lineages in the class Spirotrichea often dominate ciliate communities in open ocean waters. They have a pivotal position in marine food webs as these largely heterotrophic lineages are predators of both phytoplankton and bacteria [8–10]. Members of the class Spirotrichea have been observed in a wide range of locations: from surface to the deep ocean [10, 11], and from polar to tropical waters [9, 12]. Previous studies by morphological analysis [12–15] or by molecular approaches (e.g. HTS, DGGE) focusing on Spirotrichea [e.g. 16, 17] or all microbial eukaryotes [e.g. 5, 18] show that, within the Spirotrichea, the subclasses Oligotrichia and Choreotrichia dominate the marine planktonic ciliates. Analyses of oligotrich and choreotrich ciliates, the main focus of the current study, have found complex patterns of diversity on a large spatial scale (transect of 130 km with samples every 6 km) [16, 17]. These studies reveal that the patterns of variation in ciliate communities are related to distance from shore and to the degree of water column stratification, as evidenced both by analyses of DGGE and HTS [16, 17, 19].

The spatial scale of variation in ciliate diversity is not well known. Like many microbial eukaryotes, ciliates exhibit boom-bust cycles (i.e. rapid growth interspersed with quiescent periods) related to their relatively short generation time, the availability of pulses of nutrients, the presence of predators, and other environmental conditions [20]. A typical example of this boom-bust cycle among microbes is the phytoplankton bloom, characterized by a rapid increase in abundance of photosynthetic species (e.g. green algae, diatoms, dinoflagellates). In marine systems, phytoplankton blooms generally occur on the continental shelf at mid-latitudes during the spring and are often monospecific or nearly so [21, 22]. While phytoplankton blooms persist over large areas (100s of km^2^) for a relative long period of time (weeks) as observed by remote sensing [22, 23], short-lived blooms (days) have been described for mixotrophic ciliates such as *Mesodinium rubrum* [9, 24]. Other ciliates, such as *Lohmaniella oviformis*, have been reported to form patches of high abundance at small scales (1-10s of km^2^) [25, 26], the scale of bloom used in this study. The boom-bust cycles of protist species abundance create challenges for sampling populations adequately, and this has traditionally limited inferences about planktonic food web structure, especially in coastal waters [27].

To have a better understanding of the spatial scale of variation of oligotrich and choreotrich ciliates, we use a combination of DGGE and HTS to assess the diversity patterns at spatial scales of 1–2 km from three areas that are at least 25 km apart. We hypothesized that ciliate communities will be the similar in samples taken close together and that beyond some minimum distance, the community membership changes. We also expected to find strong assemblage changes related to the distance from shore as previously observed [16, 17, 19] such that communities are more diverse offshore. To evaluate these hypotheses, we contrasted the biogeographies of individual taxa (operational taxonomic units, or OTUs) with those of whole assemblages and evaluated patterns associated with physical and biological features of the environment.

## Materials and Methods

### Sampling

**Fig 1:**
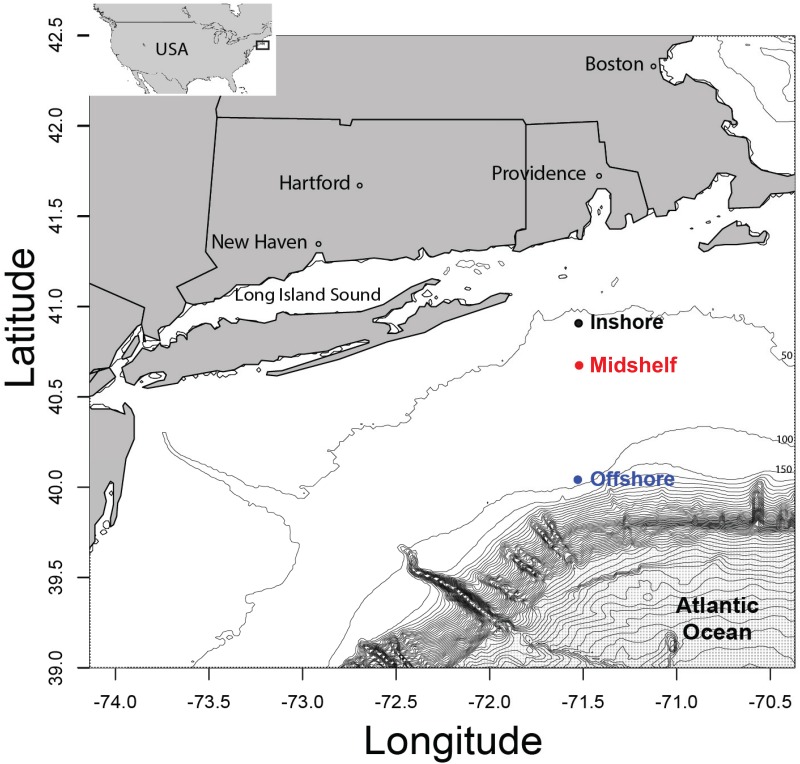
The three sampling locations on the New England shelf beyond Narragansett Bay. For each sampling location, we sampled the second station 1Km away from the first one, and the third 2Km away from the second station (see S1 Fig for more details).

We sampled three locations on board of the R/V Cape Hatteras: offshore (just before the shelf break; stations 25, 26 and 27), midshelf (stations 31, 32 and 33), and inshore (34, 35 and 36; Figs 1 and S1) from the evening of the 8^th^ to the morning of the 9^th^ July 2012 (around 1 hour between stations during the 12 hour transit inshore from the shelf break). No permits were required for the field sites as the locations are neither privately-owned nor protected, and the studied organisms did not include endangered species. At each location, the first two stations were separated by 1 km, and the third station was 2 km away from the second station (S1 Fig). Three depths were sampled: surface, chlorophyll maximum depth, and deep (c. 5 m above the seafloor) layers (see S1 Fig). A CTD profiler (Sea-Bird Electronics, Inc., WA) mounted on a rosette measured the temperature, salinity, chlorophyll fluorescence and oxygen at each station.

### Sample filtration and DNA extraction

For each DNA sample, one liter of seawater was collected by Niskin bottle. The collected seawater was then prescreened through 80 μm nylon mesh to remove large organisms and associated potential PCR inhibitors, and filtered in series onto 10μm and 2μm polycarbonate filters to separate the micro- and nanosize fractions. The filters were immediately placed into 0.5 mL of DNA preparation buffer (100 mM NaCl, Tris-EDTA at pH 8, and 0.5% SDS) and stored at 4°C until DNA extraction. Our sampling protocol resulted in 54 samples: 3 sampling locations, each with 3 stations, times 3 depths, times 2 size fractions. For extraction, we used a modified phenol-chloroform method [28].

### DNA amplification

The DNA from the filters was amplified under conditions aimed at minimizing PCR recombination artifacts. The amplifications were carried out using primers specific to ciliates within the Spirotrichea class (152+ and 528-, OCSP-A from [29, 30]), which target the hypervariable V2 of the SSU rDNA. For each amplification, twenty microliters of master mix was used [4 μL 5xGC buffer (NEB, MA), 0.5 mM of MgCl, 50 mM of bovine serum albumin, 50 μM of each dNTP, 0.25 pM of each primers, and 0.1 μL Phusion polymerase (NEB, MA)] under the following cycling conditions: 98°C for 3 min, 30 x (98°C for 15 s, 58°C for 15 s, 72°C for 1 min) and final extension at 72°C for 10 min. In order to reduce PCR bias, five independent PCR products per sample, using a 1/10 dilution of genomic DNA, were pooled together for denaturant gradient gel electrophoresis (DGGE) and for high-throughput sequencing (HTS), using the appropriate primer sets (see below) [31].

### Denaturant gradient gel electrophoresis analysis

A 39 base length GC clamp (CGC CCG CCG CGC CCC GCG CCC GTC CCG CCG CCC CCG CCC) was added to the reverse primer for DGGE. Each depth layer and size fraction (one gel for surface, chlorophyll maximum and deep layer and for 2 and 10μm DNA samples i.e. a total of 6 gels) were run on an independent DGGE gel—i.e. each gel had six stations (25, 26, 27, 31, 32, 33, 34, 35 and 36). Using the Dcode Universal Mutation System (Bio-Rad, CA) DGGE setup, 6% acrylamide gels with a denaturant gradient from 35 to 55% (100% denaturant corresponds to 7 M urea and 40% deionized formamide) were run at 245 V for 5 minutes followed by an incubation at 45 V for at least 15 hours. Gels were stained for 30 min in 200 mL of TAE buffer with 20 μL of SYBR Gold (Invitrogen, CA) and photographed using Kodak molecular imaging software (Carestream Health, Inc. NY). We previously confirmed the robustness of our DGGE primers and methods by replicating several gels using PCR reactions run on different days [17, 32].

### High-throughput sequencing analysis

Adaptors for multiplexed 454 sequencing were added to our Spirotrichea primers for HTS. Emulsion PCR and 454 sequencing (pyrosequencing by synthesis) followed standard protocols for the GS FLX Titanium instrument (454 Life Sciences, Roche, Branford, CT, USA). The raw reads are available under the BioProject PRJNA339757 and Sequence Read Archive SRP082687. We analyzed the data following recommendations from previous studies [16, 19, 33, 34]. In summary, OTU libraries were built in QIIME version 1.9.1 [35], clustering with Uclust 1.2.22q at 99% similarity [36]. OTUs with fewer than 5 reads, chimeras identified by Uchime_denovo v4.2.40 [37], OTUs with ambiguous bases, and out-group sequences were discarded to remove noise resulting from HTS. On 54 samples that had fewer than 100 total reads, we discarded OTUs with <4 reads. Starting with 178,711 reads (101 to 13,476 reads per sample), we obtained 782 OTUs (3 to 129 OTUs per sample). We pooled the sequences from the two size fractions (10–80 μm micro-ciliates and 2–10 μm nano-ciliates) to create a third dataset to analyze spatial distributions for the entire sample. To evaluate geographical and vertical patterns, we randomly selected 100 or 500 reads (200 or 1,000 for the pooled size fraction dataset) for each sample as this allowed us to normalize the output and avoid bias due to varying numbers of reads per sample. This approach resulted in 192 OTUs (1 to 31 per sample) when subsampling 100 reads. At 500 reads, we had to discard 13 samples (those with fewer than 500 reads). The 37 remaining samples varied in containing 1 to 53 OTUs.

### Taxonomic assignment for DGGE phylotypes and HTS OTUs

For DGGE, the brightest bands (i.e. phylotypes) and all common phylotypes were excised from the gels, amplified by 10 cycles of PCR, and sequenced by the Sanger method (GenBank accession numbers KX714479-KX714518). The resulting phylotypes from DGGE and the OTUs from HTS were compared to our curated list of morphospecies sequences from GenBank and DGGE phylotypes from previous studies [16, 17, 19]. We used two approaches to assign taxonomy: a BLAST approach and a phylogenetic approach. For the BLAST approach, we kept only matches with the full length of our OTUs, and with a similarity of 99% or an E-value > 2e^-150^. For the phylogenetic approach, we aligned our sequences with Mafft E [38] and built phylogenetic trees using RAxML v8.2.4 [39] with the Evolutionary Placement Algorithm implemented in RAxML (EPA, [40]). OTUs and phylotypes were then identified by position on the trees.

### Statistical analyses and sample clustering

We used EstimateS v9 [41] to calculate OTU richness, along with the Shannon’s index (H’; [42, 43]) and Chao1 estimator (S_chao1_; [44]) for diversity. We used H’ to estimate the number of OTUs that are major contributors to community structure. S_chao1_ was used to estimate the ‘maximal’ or expected total diversity in each sample. Dissimilarity matrices were calculated using Fast Unifrac [45] (unweighted Unifrac metric: difference between samples based on OTU composition and a gene tree) from phyloseq v1.16.2 in R v3.2.3 [46] and for performing principal coordinates analysis (PCoA). We used the Mahalanobis distance metric to cluster samples based on environmental parameters. Canonical correlation analysis (CCA) was also performed to assess relationships between environmental parameters, community and OTUs composition. Analysis of similarities (ANOSIM; vegan package 2.4–1 in R) was used to test the difference between groups of samples (i.e. clusters), with 999 permutations.

## Results

### Environmental parameters

**Fig 2:**
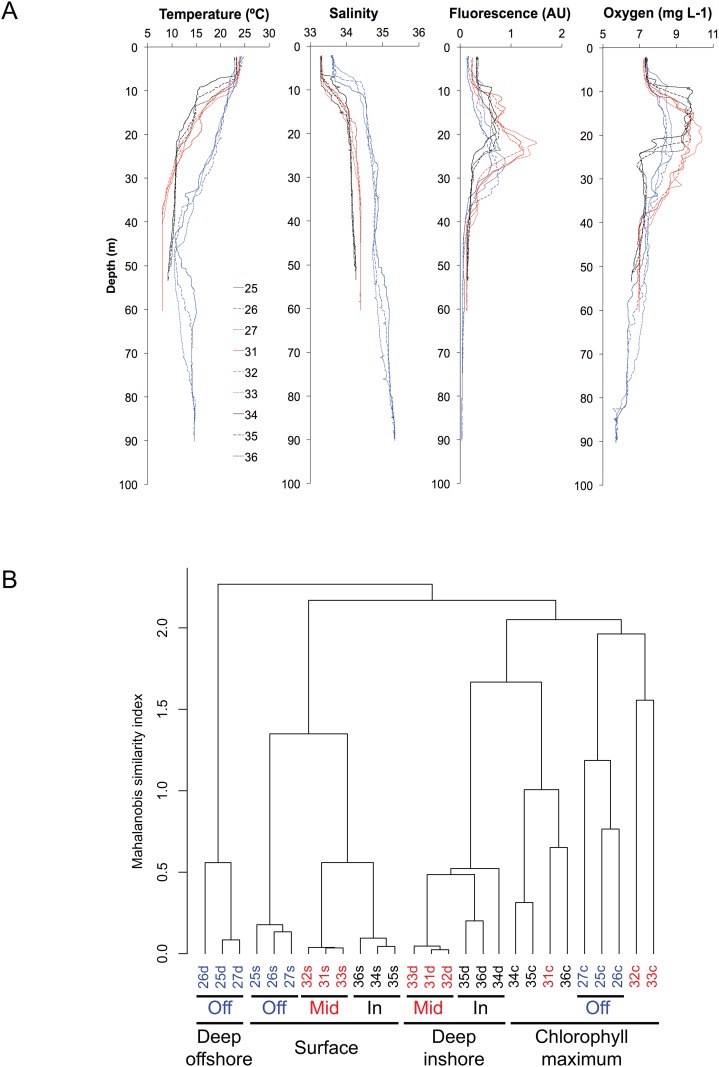
Analyses of environmental parameters. A. Environmental parameters show the separation with the three sampling locations (blue offshore, red midshore and black inshore). B. Sample clustering by environmental parameters using Mahalanobis distance metric in R [46] shows similarity by layer (Surface, Chlorophyll maximum and deep) and then by position on the shore (inshore, midshelf and offshore).

Environmental parameters (temperature, fluorescence, salinity, and dissolved oxygen) varied mainly by depth layer and distance from shore (Fig 2A). All surface samples formed a single cluster in the Mahalanobis distance metric, as did the midshelf and inshore deep samples; the offshore deep samples formed a separate, quite distinct cluster (Fig 2B). Chlorophyll maximum samples were split, with the three offshore stations clustering together with two midshelf stations, and the third midshelf station clustering with the inshore stations (Fig 2B).

### Community composition by DGGE

**Fig 3:**
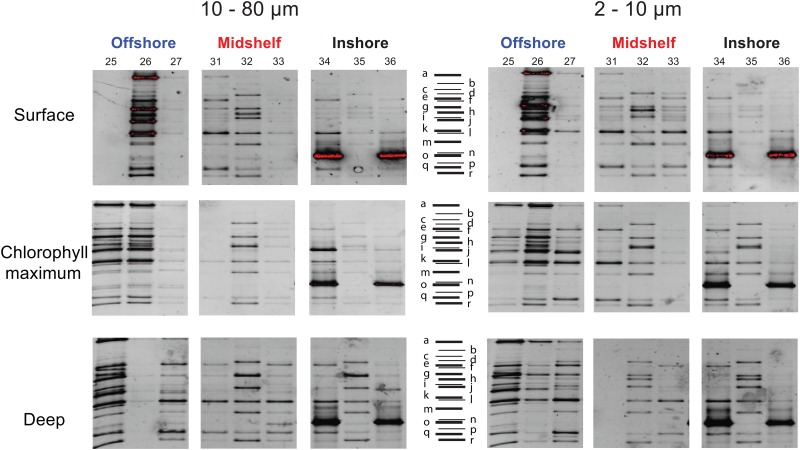
Analyses of common community members by DGGE reveal a more diverse community offshore at every depth, and patchiness for the midshore and inshore stations. The middle station of the inshore and midshore has a different community of abundant taxa as compare to the two other stations. A bloom by one OTU is evidence by DGGE at stations 34 and 36. Empty lanes represent samples that failed to PCR, likely due to either presence of PCR inhibitors in the water column or the absence of target community members.

DGGE shows patterns for the abundant community members. We observed some variation in brightness and some empty lanes, as some samples did not amplify robustly (Fig 3), so here we consider only presence/absence of different phylotypes. We did not detect strong differences in community composition between the two size fractions. Altogether, we observed 23 unique phylotypes and 13 phylotypes that were shared between at least 2 samples (S3 Fig). Offshore stations were the most diverse in terms of numbers of phylotypes (15 to 21 phylotypes), while the inshore and midshelf stations were more similar (9 to 12 phylotypes; Fig 3; S1 Table). The offshore stations show a slight increase in the number of phylotypes with depth, but overall the communities are similar throughout the water column (Fig 3). At a smaller spatial scale (i.e. within each location), communities were similar among stations. At both the inshore and midshelf locations, the middle station showed a different pattern from the 'edge' stations on either side, which were similar to one another. We interpret this as evidence of patchiness on a scale of 1–3 km (distance between stations). For example, midshelf stations 31 and 33 share a similar band pattern while station 32 shares some phylotypes but overall also has some unique phylotypes (Fig 3). The same pattern is observed for the inshore location; the samples of the two edge stations look more similar compared to the middle station, which shares only a few phylotypes through the water column with its neighboring stations. We also found that the edge stations of the inshore location clearly show the dominance of one phylotype (illustrated in red for stations 34 and 35 surface; Fig 3). This phylotype (phylotypes marked 'o' from sample 34d2, 36d10, 36s2, and 34c10, respectively; Figs 3 and S3) is closely related to the choreotrich *Pelagostrobilidium paraepacrum* (FJ876963; 95% identity; S2 Table).

### Community composition by HTS

Although the community composition varies by size fraction, location and depth, the number of OTU do not show a clear pattern (S1 Fig). In fact, the diversity estimates (i.e. total number of OTUs, Shannon (H’) and Chao1 indices) are relatively similar across size fractions and depths; for example, the total number of OTUs subsampled at 100 reads ranges from 1 to 26 and 1 to 31 for the nanosize and microsize, respectively (S1 Fig; S1 Table). By contrast, spirotrich community composition changes across size fractions and depths (S2 Fig). For example, the microsize fraction of the offshore stations is dominated by the second most abundant taxon (OTU2165) in the chlorophyll maximum and deep samples, but the same OTU is almost absent from the surface layer and in the nanosize fraction. This OTU is 99.4% identical to the tintinnid *Salpingella acuminata* (EU399536; S2 Table). Similarly, the third most abundant taxon (OTU2594) is identical to *Strombidium cf*. *basimorphum* (JF791016; S2 Table) and is very abundant in the nanosize fraction, particularly the surface and chlorophyll maximum of the offshore stations; by contrast OTU2594 is almost absent in the deep samples and in the microsize fraction (S2 Fig). The HTS data also show that the edge stations of the inshore location are dominated by one OTU throughout the water column, as observed with DGGE. Again, this taxon (OTU329) is closely related to the choreotrich *Pelagostrobilidium paraepacrum* (95% identity) and is identical to the sequence observed with DGGE (phylotypes marked with 'o' in S3 Fig).

**Fig 4:**
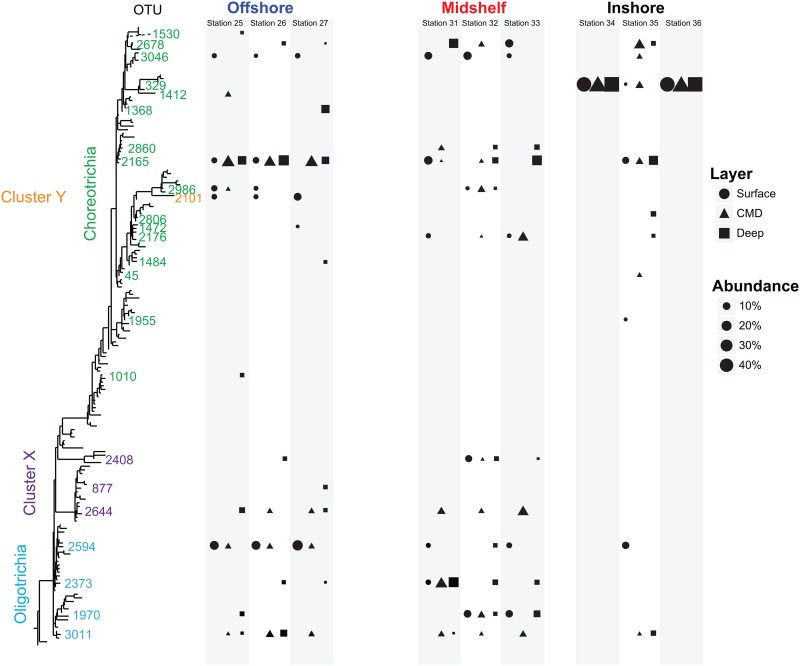
Heterogeneous patterns of OTUs (i.e. OTU specific to the inshore or to the offshore area, to the photic zone or to the deep) mapped onto phylogenetic tree. Only OTUs representing more than 5% of the communities were considered to observe the overall patterns. The bloom ciliate, OTU329 is evident in Stations 34 and 36.

To observe the taxonomic pattern at varying scales, we mapped patterns of abundant OTUs (> 5% of all reads) onto a phylogenetic tree (Fig 4). At the inshore location, OTU329 (*Pelagostrobilidium paraepacrum*-like) dominated at stations 34 and 36, comprising nearly 100% of the read numbers throughout the water column. In contrast, the intervening station 35 is composed from 4 to 6 abundant taxa, including OTU329. We also found some OTUs whose distribution varied by depth layer rather than location. For example, while the OTU2165 is observed throughout the water column, OTU3046 and OTU2101 are present only at the surface. Other taxa are present only below the photic zone such as the OTU2594, while two taxa (OTU2644 and OTU3011, Fig 4) seem to be specific to the chlorophyll maximum layer. Overall, we observed that (1) 51 OTUs are specific to the nanosize fraction, 68 to the microsize fraction and 50 are in both sizes; and (2) a core community composed of 23 OTUs shared across locations and/or depths while the majority of OTUs are specific to a location (inshore, midshelf or offshore) and/or to a layer (surface, chlorophyll maximum and deep; S4 Fig).

### Community biogeography and environmental parameters

**Fig 5:**
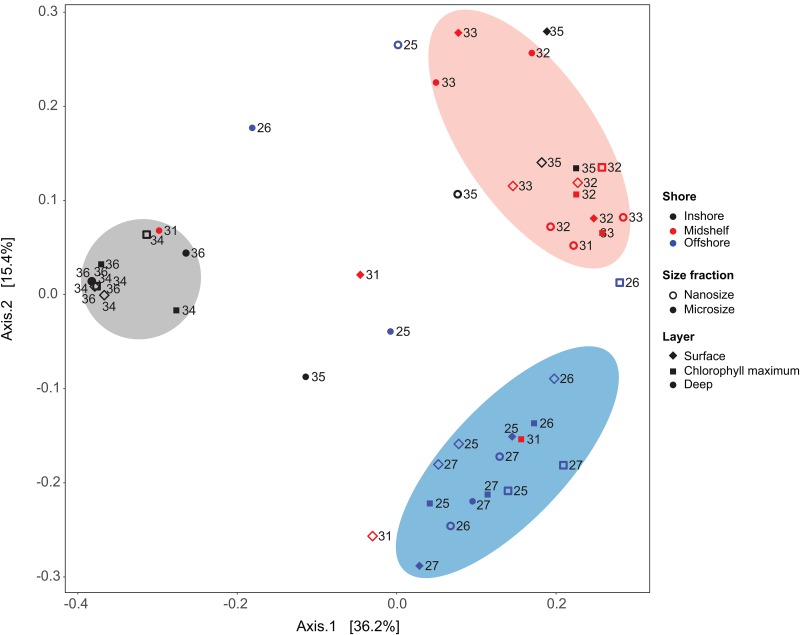
Principal coordinate analyses using Unifrac dissimilarity metric (HTS data) reveals three clusters emerge that generally correspond with inshore (gray), midshelf (red) and offshore (blue) samples. The clusters show some overlap between midshelf and offshore (red symbols within the blue cluster), and between the midshelf and inshore (red within the gray cluster), but little overlap between inshore and offshore.

Clustering analysis using Unifrac unweighted distance and principal coordinate analyses (PCoA) discriminates assemblages (clusters of sample sharing a similar community) by location (ANOSIM R = 0.09, p < 0.05; Fig 5). Samples generally clustered based on their distance from shore, with the inshore or ‘bloom’ stations (stations 34 and 36) clustering together across all depths, and two other clusters corresponding to the midshelf and offshore stations. The ciliate communities do not cluster by depth even though the environmental parameters do (Fig 2). Canonical correlation analysis shows that temperature and salinity are the major abiotic factors, but do not show clear relationships with the communities or the OTUs (S5 Fig). Examining the clustering at each location separately (S6 Fig), we did not find any patterns: 1) there is no evidence of further clustering within the offshore and midshelf samples (blue and red clusters in Fig 5); and 2) the two samples in bloom at the inshore location (e.g. 34 and 36) are distinct from the intervening station (35; S6 Fig).

## Discussion

Comparisons of HTS and DGGE data from samples taken at three locations (inshore, midshelf and offshore) off the New England coast lead to two main insights: 1) ciliate communities are patchily distributed on the shelf at scales of 1–3 km; and 2) ciliate communities and abundant OTUs do not share the same biogeographies.

### Patchiness varies from shelf to offshore

Ciliate communities show a complex distribution related to their distance from shore and from each other. Communities sampled across large spatial scale (inshore, midshelf and offshore) clustered by location but did not show any clear clustering at small spatial scales (i.e. 1, 2, or 3 km from one another) when sampled offshore and midshelf while communities are patchier at the same spatial scales when sampled at inshore stations (S5 Fig). The same observation is made by DGGE or HTS; DGGE OTUs represent the most abundant HTS OTUs (S3 Fig), which is consistent with previous findings [16]. This suggests that distance to the shore has a more powerful role in structuring ciliate assemblages than specific features of the environment (temperature, salinity, oxygen, or phytoplankton biomass) do. The similarities in community composition at the offshore stations are related to their distance from one another as assessed by both DGGE and HTS (Figs 3–5, S2 and S6). These observations are congruent with previous studies [4, 16, 17] though at a finer scale, suggesting a link with phytoplankton, predator distributions, or small-scale physical processes [19].

By contrast, the midshelf and inshore ciliate communities are patchily distributed with communities at the middle station distinct from its two neighboring stations (Figs 3, 4 and S6). This patchiness is most apparent at the inshore location where we observed a bloom throughout the water column of a ciliate (OTU329 or phylotype o) that is closely related to *Pelagostrobilidium paraepacrum* (Figs 3–5 and S3). This OTU dominates in DGGE (phylotype o; Fig 3) and HTS (OTU329; Figs 4 and S2) in both the nanosize (2–10 μm) and in the microsize (10–80 μm) fractions at stations 34 and 36, but is relatively rare at the intervening station 35 (0–30% of HTS reads and no clear band on DGGE; Fig 2). While eddies with a strong salinity and temperature signature have been observed at this small scale in the New England shelf [47–49], suggesting a physical mechanism driving patchiness, we did not detect any clear difference within the temperature and salinity between the middle station and the two edge stations where we observed this OTU (Fig 2). In other words, this bloom is not obviously related to the environmental parameters we measured as salinity, temperature, oxygen and fluorescence change strongly with depth but not distance from shore at these stations (i.e. stations 34–36; Fig 2A). The fact that the bloom was observed from surface to deep suggests that environmental conditions are not the driving factors, instead suggesting that the bloom is either driven by a biotic factor (e.g. the presence of a particular predator or prey), a property of water circulation that we did not measure, or is stochastic.

Monospecific blooms in marine systems are well-known, particularly for phytoplankton such as diatoms [50], dinoflagellates [51] and haptophytes [52]. Temperature and nutrients (e.g. nitrate, phosphate and silicate) are the main initiators of phytoplankton blooms, but low planktonic grazing activity compared to phytoplankton growth allows their expansion [14, 53, 54]. Some ciliates such as *Lohmaniella oviformis* and *Mesodinium rubrum* (= *Myrionecta rubra*) are known to bloom in small-scale patches (13–170 m) and throughout the water column [24–26]. These blooms have been argued by some authors to be related to the patchiness of the phytoplankton prey [26, 55] while others suggest a relation to the short generation times of small ciliates and their aggregative swimming behavior [25], physical processes, or a combination of all these [43].

### Biogeography: Community vs individual taxa

Ciliate communities clustered distinctly based on distance from shore (i.e. in PCoA analyses on presence/absence data, Fig 5). However, within each sampling location, PCoA shows variable clustering (patchiness) apparently not linked to depth. In contrast with the environmental factors that appear to vary mostly by depth (Fig 2A), clustering of ciliate communities does not correspond to depth through water column (Fig 5). The absence of relationships with the environmental parameters and a strong correlation between community composition and position on the shelf have been observed in our previous studies on a larger scale with more evenly distributed stations, including all Oligotrichia plus Choreotrichia community members or just the morphologically well known tintinnid choreotrichs [16, 17, 19].

Other studies show patterns of the community composition for specific clades or all protists assessed by HTS related to depth, oceanic basin or time (e.g. [4–6, 56]). For example, Massana et al [5] show protist distributions related to geography and time at six European coastal sites but did not find relationships with any environmental parameters. In the same way, de Vargas et al [4] show a distribution of communities by size fraction and by oceanic basin, with distributions in ecological groupings but nothing related to abiotic factors. Bittner et al [56] show a relationship between haptophyte community composition and depth but did not link this pattern to any environmental parameters.

By contrast, individual taxa (i.e. OTUs) show specific biogeographies related to depth layers that are not observed at the whole-community level. These distinct biogeographies are best illustrated by Venn diagrams (S4 Fig) and by mapping the distribution of OTUs on a phylogenetic tree (Fig 4). Some taxa (e.g. OTU2165) are widespread, while others are isolated by distance (e.g. OTU2860) or layer (e.g. OTU3046). The presence of OTU2594 only within the photic zone suggests a role of light, which affects both mixotrophic ciliates such as *Strombidium chlorophyllum* and phytoplankton grazers such as *S*. *siculum*. Other taxa (e.g. OTU1970) have complex patterns that may be related either to parameters not measured here or they may reflect neutral processes [57, 58]. Our observations are consistent with "the paradox of the plankton" [58, 59], as OTUs that appear to occupy the same ecological niche do not seem to be subject to competitive exclusion and instead they coexist.

## Synthesis

We used DGGE and HTS to quantify patterns of ciliate community composition across the New England continental shelf. The most important insight of our study is that the biogeographic patterns of communities and individual taxa (i.e. OTUs or phylotypes) appear to be decoupled: whole communities vary by distance from the shore, while the distributions of individual lineages are more closely linked to depth strata. This suggests that to understand the distribution and ecology of communities, we need to have a better knowledge of each member of the community, including the rare species that are only seen in HTS.

## Supporting Information

S1 Fig Analyses of estimated diversity from inshore to offshore and by depth reveal no clear pattern and few differences between nanosize (2–10μm) and microsize (10–80μm). A total of 100 (11±8 for nanosize and microsize) and 500 reads (20±13 and 24±16 OTUs for the nanosize and microsize, respectively) was subsampled for A and B, respectively. The diversity indices show the same pattern using H’ index (C, from 0.005 to 3.04, 1.5±0.9 for the nanosize and the microsize) and the Choa1 diversity estimator (D, from 3 to 232; 49.7±54.5 and 57.1±54.2 for the the nanosize and the microsize, respectively).
(DOCX)

S2 Fig Ciliate communities composition (percentage of reads) for abundant OTUs (more than 5% of the total number of reads) at 99% ID shows constancy in pattern within the offshore stations compared to the midshelf and inshore stations. In contrast, there is evidence of a bloom at all depths in two non-adjacent inshore samples (i.e. stations 34 and 36). Each OTU has an unique identifier, follow by the number of reads and occurrence of this OTU during the cruise (this study and Grattepanche et al, 2016), followed by the best BLAST result (S2 Table).
(DOCX)

S3 Fig Phylogenetic tree shows the similarity between OTUs obtained by DGGE and HTS. In black, HTS OTUs with more than 1,000 reads were considered to simplify the tree. In orange are the OTUs from DGGE analyses (See Fig 3). OTU with an asterisk were not represented in Fig 4.
(DOCX)

S4 Fig Canonical correlation analysis shows only that salinity and temperature are major abiotic features across sites. (DOCX)

S5 Fig Principal coordinate analyses using Fast Unifrac dissimilarity metric do not show clear clustering of Ciliates community composition at small scale (1, 2, and 3 km) for the offshore and midshelf. (DOCX)

S6 Fig Venn diagrams show specificity of OTUs for a size, a position on the shore and/or for a layer, but also the presence of a core community (23 OTUs) in all our samples. (DOCX)

S1 Table Reads, OTU numbers (with or without subsampling) and diversity indices (See S1 Fig). (DOCX)

S2 Table OTU best BLAST results to a morphospecies (see S2 Fig). (DOCX)

S3 Table Pearson correlations show complex relationship between OTUs and environmental parameters. Some abundant OTUs are related to the distance to the shore and to the depth (e.g. OTU329 mainly observed inshore or OTU2594 mainly observed within the surface layers in the offshore location) as observed in Fig 4.
(DOCX)
