# Supplementary material for: Patchiness of Ciliate Communities Sampled at Varying Spatial Scales along the New England Shelf
Source: PLoS One. 2016 Dec 9;11(12):e0167659. doi: 10.1371/journal.pone.0167659 (PMC5147948; doi:10.1371/journal.pone.0167659)
Supplement: S1 Fig — A total of 100 (11±8 for nanosize and microsize) and 500 reads (20±13 and 24±16 OTUs for the nanosize and microsize, respectively) was subsampled for A and B, respectively. The diversity indices show the same pattern using H’ index (C, from 0.005 to 3.04, 1.5±0.9 for the nanosize and the microsize) and the Choa1 diversity estimator (D, from 3 to 232; 49.7±54.5 and 57.1±54.2 for the the nanosize and the microsize, respectively). (DOCX) [file pone.0167659.s001.docx]

**S1 Fig.** Analyses of estimated diversity from inshore to offshore and by depth reveal no clear pattern and few differences between nanosize (2-10µm) and microsize (10-80µm). A total of 100 and 500 reads was subsampled for A and B, respectively. The diversity indices show the same pattern using H’ index (C) and the Choa1 diversity estimator (D) (Table S1).

**Offshore Midshelf Inshore**

25 26 27 31 32 33 34 35 36

1Km 2Km 65Km 1Km 2Km 27Km 1Km 2Km

| **A.** |  |
| --- | --- |
| **B.** |  |
| **C.** |  |
| **D.** |  |

Note: for stations 31, 33 and 35, the chlorophyll maximum data are not available for the nanosize fractions. For station 26, the microsize fraction is not available. The 100 reads subsampling was used to have a better picture and avoid having too much gaps. Number used for this figure are in Table S1
