# Supplementary material for: Patchiness of Ciliate Communities Sampled at Varying Spatial Scales along the New England Shelf
Source: PLoS One. 2016 Dec 9;11(12):e0167659. doi: 10.1371/journal.pone.0167659 (PMC5147948; doi:10.1371/journal.pone.0167659)

**S2 Fig.** Ciliate community composition (from 100 reads subsampling) shows constancy in pattern within the offshore stations compared to the midshelf and inshore stations. In contrast, there is evidence of a bloom at all depths in two non-adjacent inshore samples (i.e. stations 34 and 36).

2 -10µm 10 - 80µm

**Offshore Midshelf Inshore Offshore Midshelf Inshore**


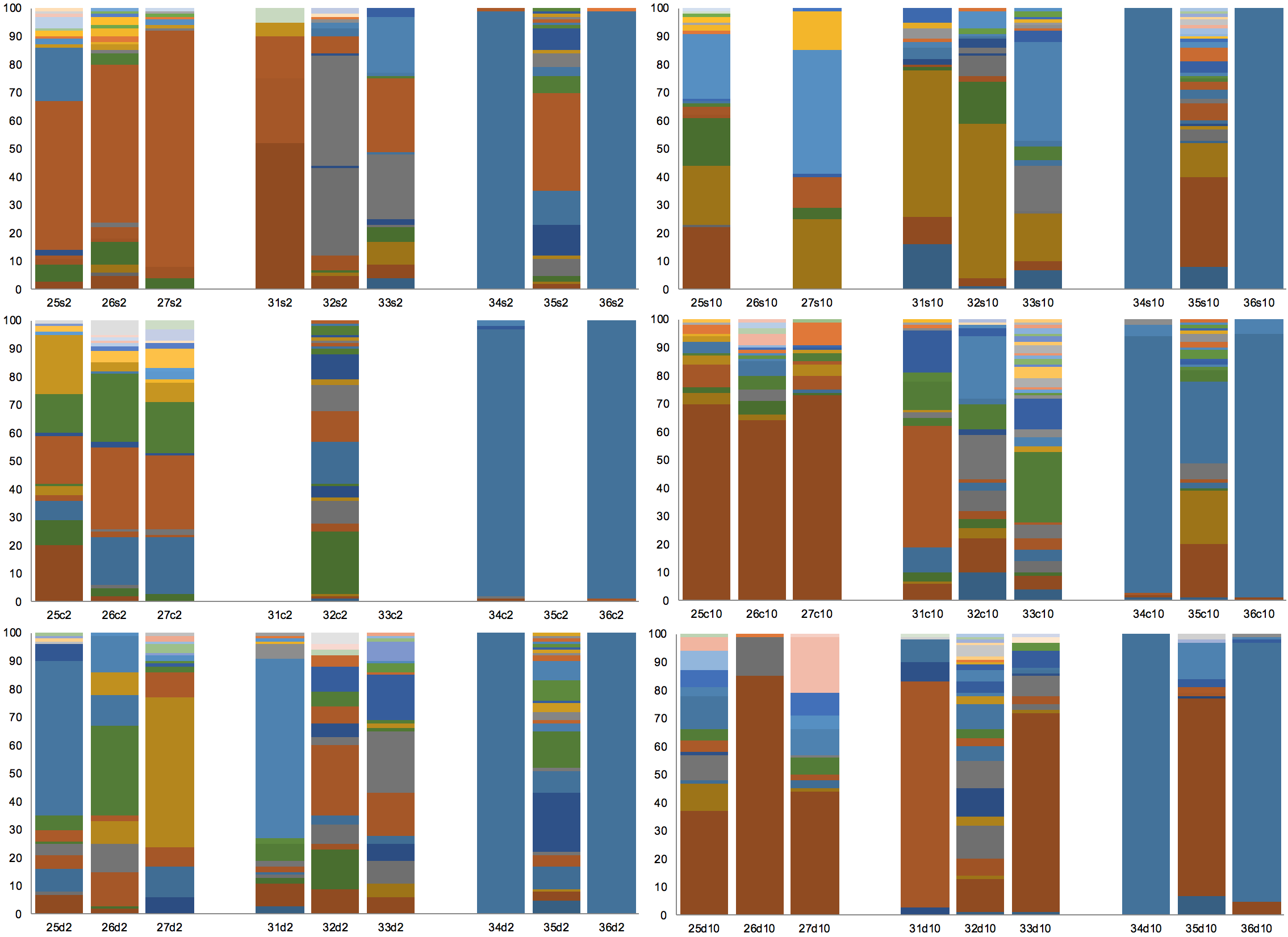


Surface

Chlorophyll maximum

Deep


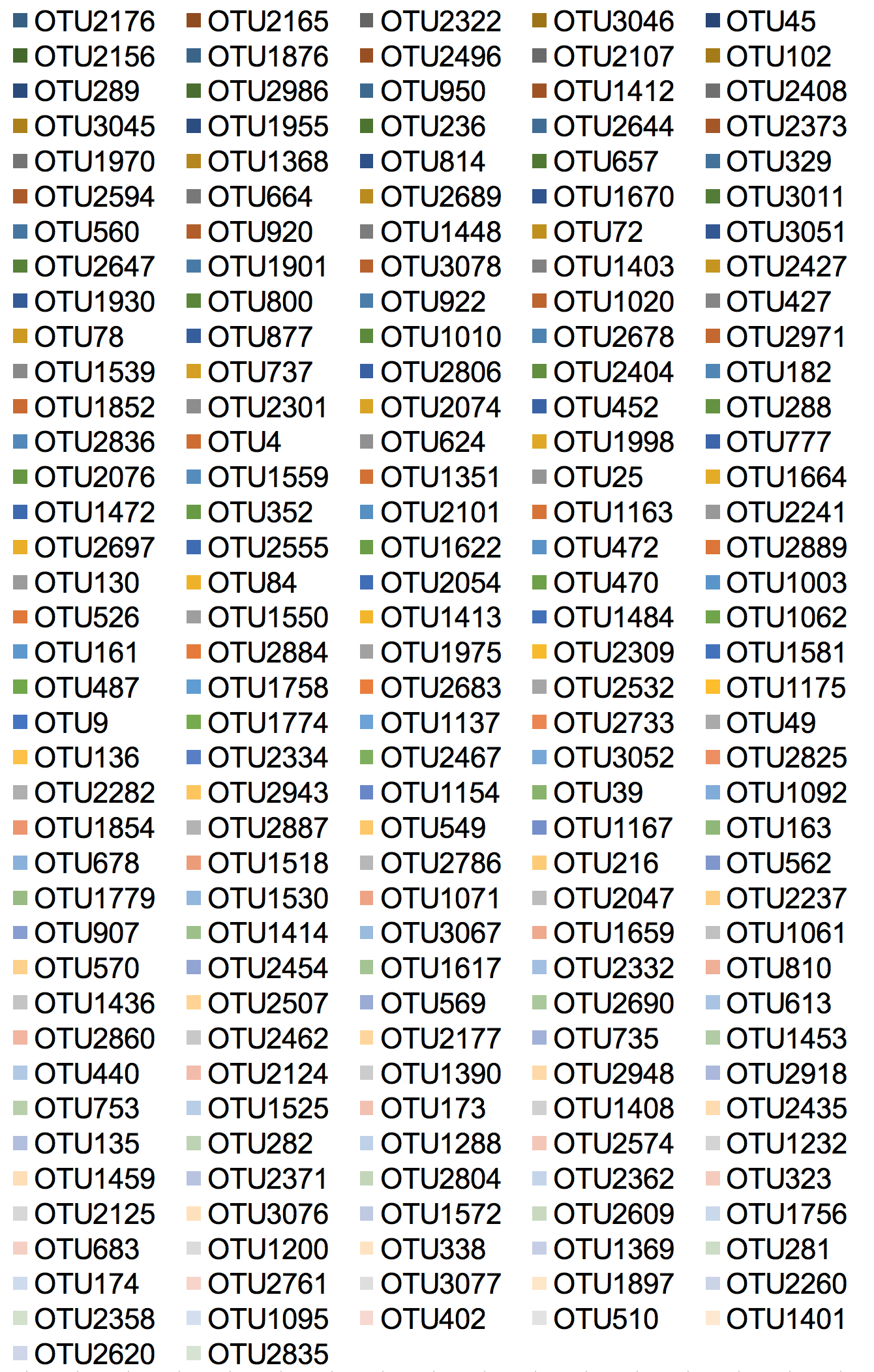

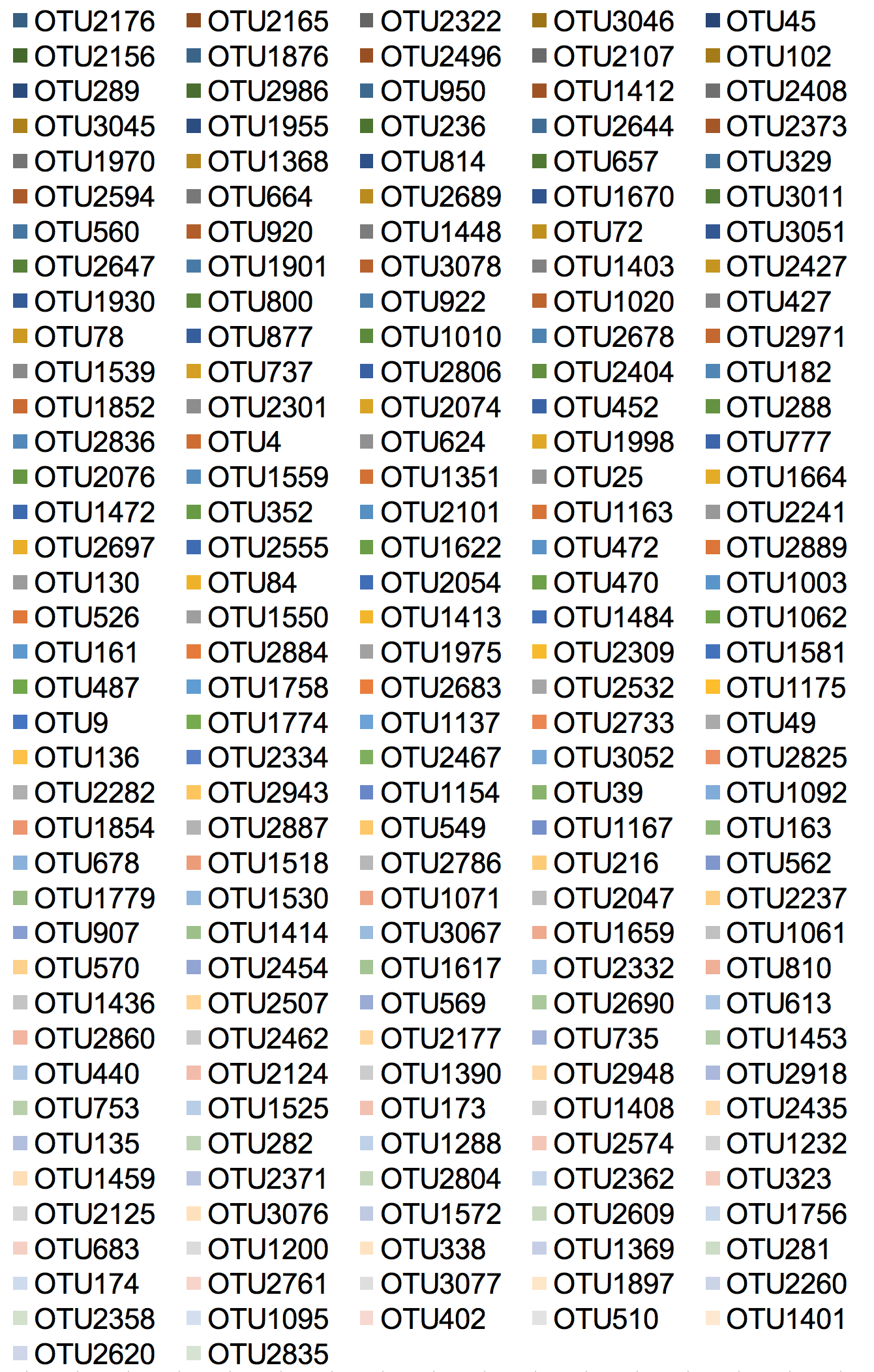

Supplement: S2 Fig — In contrast, there is evidence of a bloom at all depths in two non-adjacent inshore samples (i.e. stations 34 and 36). Each OTU has an unique identifier, follow by the number of reads and occurrence of this OTU during the cruise (this study and Grattepanche et al, 2016), followed by the best BLAST result (S2 Table). (DOCX) [file pone.0167659.s002.docx]
