# Supplementary material for: Patchiness of Ciliate Communities Sampled at Varying Spatial Scales along the New England Shelf
Source: PLoS One. 2016 Dec 9;11(12):e0167659. doi: 10.1371/journal.pone.0167659 (PMC5147948; doi:10.1371/journal.pone.0167659)

**S3 Fig.** Phylogenetic tree shows the similarity between OTUs obtained by DGGE and HTS. In black, HTS OTUs with more than 1,000 reads were considered to simplify the tree. In orange are the OTUs from DGGE analyses (See Figure 3). OTU with an asterisk were not represented in Fig. 4


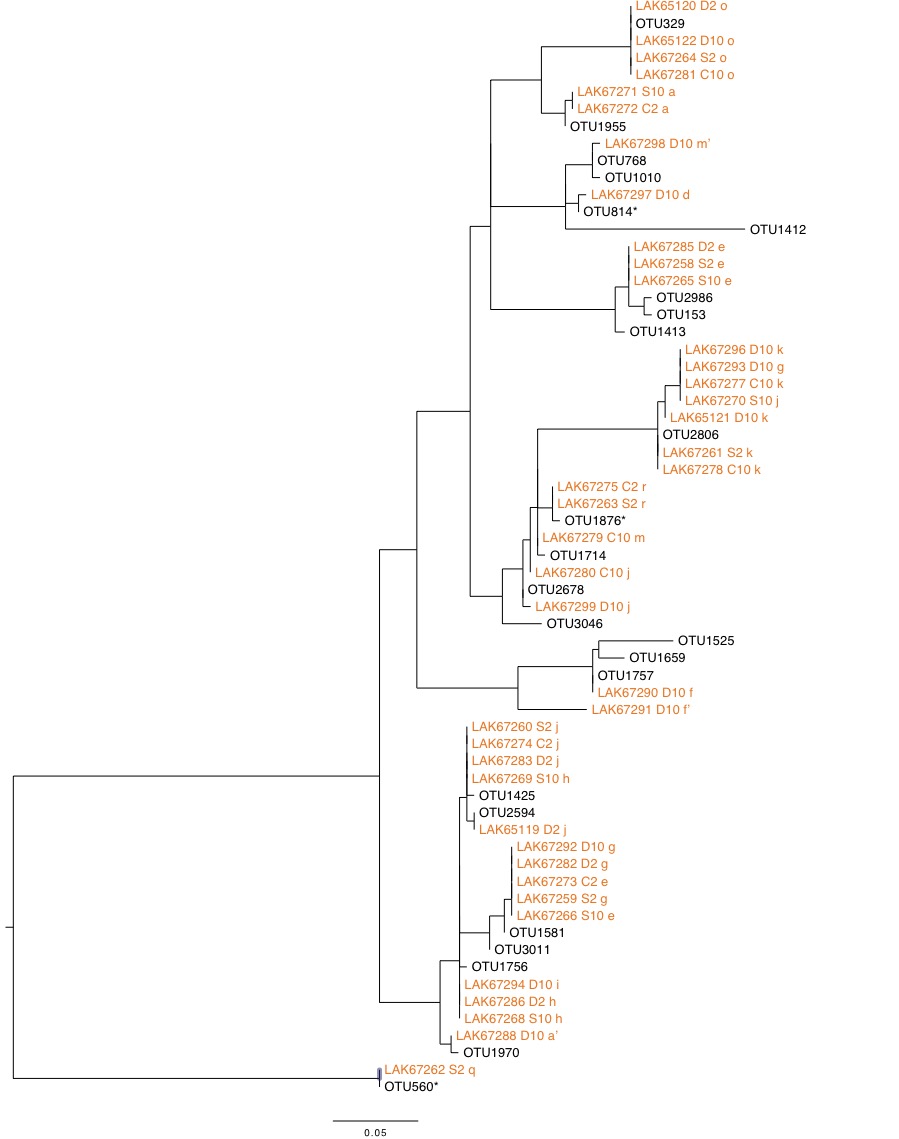

Supplement: S3 Fig — In black, HTS OTUs with more than 1,000 reads were considered to simplify the tree. In orange are the OTUs from DGGE analyses (See Fig 3). OTU with an asterisk were not represented in Fig 4. (DOCX) [file pone.0167659.s003.docx]
