# Supplementary material for: Patchiness of Ciliate Communities Sampled at Varying Spatial Scales along the New England Shelf
Source: PLoS One. 2016 Dec 9;11(12):e0167659. doi: 10.1371/journal.pone.0167659 (PMC5147948; doi:10.1371/journal.pone.0167659)
Supplement: S4 Fig — (DOCX) [file pone.0167659.s004.docx]

**S4 Fig.** Venn diagrams show specificity of OTUs for a size, a position on the shore and/or for a layer, but also the presence of a core community (23 OTUs) in all our samples.

|  |
| --- |
|  |
|  |
