# Supplementary material for: Patchiness of Ciliate Communities Sampled at Varying Spatial Scales along the New England Shelf
Source: PLoS One. 2016 Dec 9;11(12):e0167659. doi: 10.1371/journal.pone.0167659 (PMC5147948; doi:10.1371/journal.pone.0167659)
Supplement: S5 Fig — (DOCX) [file pone.0167659.s005.docx]

**S5 Fig.** Canonical correlation analysis shows only that salinity and temperature are major abiotic features across our sites.
