# Supplementary material for: Patchiness of Ciliate Communities Sampled at Varying Spatial Scales along the New England Shelf
Source: PLoS One. 2016 Dec 9;11(12):e0167659. doi: 10.1371/journal.pone.0167659 (PMC5147948; doi:10.1371/journal.pone.0167659)
Supplement: S6 Fig — (DOCX) [file pone.0167659.s006.docx]

**S6 Fig.** Principal coordinate analyses using Unifrac dissimilarity metric show the relationship of Ciliates community composition to the position to the shore.

**Offshore**

**Midshelf**

**Inshore**
