# Supplementary material for: Patchiness of Ciliate Communities Sampled at Varying Spatial Scales along the New England Shelf
Source: PLoS One. 2016 Dec 9;11(12):e0167659. doi: 10.1371/journal.pone.0167659 (PMC5147948; doi:10.1371/journal.pone.0167659)
Supplement: S1 Table — (DOCX) [file pone.0167659.s007.docx]

**S1 Table:** Reads, OTU numbers (with or without subsampling) and diversity indices

| sample | Reads | HTS OTU  All reads | HTS OTU  500 reads | HTS OTU  100 reads | Shannon | SChao1 | Simpson inverted | DGGE OTU |
| --- | --- | --- | --- | --- | --- | --- | --- | --- |
| 25s2 | 697 | 30 | 25 | 16 | 1.75 | 32.14 | 3.28 |  |
| 26s2 | 6726 | 76 | 32 | 19 | 1.89 | 130.17 | 2.75 | 17 |
| 27s2 | 185 | 12 |  | 10 | 0.84 | 12.75 | 1.45 | 9 |
| 31s2 | 2637 | 12 | 8 | 5 | 1.36 | 22.00 | 3.15 | 12 |
| 32s2 | 471 | 32 |  | 15 | 1.83 | 62.00 | 3.58 | 10 |
| 33s2 | 4101 | 34 | 19 | 13 | 2.03 | 49.00 | 5.43 | 10 |
| 34s2 | 567 | 6 | 6 | 2 | 0.06 | 16.00 | 1.02 | 12 |
| 35s2 | 6571 | 129 | 43 | 20 | 2.61 | 176.83 | 5.88 | 7 |
| 36s2 | 1229 | 5 | 3 | 2 | 0.03 | 11.00 | 1.01 | 9 |
| 25c2 | 159 | 17 |  | 14 | 2.15 | 31.00 | 6.77 | 12 |
| 26c2 | 959 | 32 | 27 | 18 | 2.35 | 32.67 | 6.55 | 18 |
| 27c2 | 6479 | 50 | 29 | 16 | 2.44 | 95.50 | 7.66 | 9 |
| 31c2 | nd |  |  |  |  |  |  | 10 |
| 32c2 | 11387 | 122 | 39 | 23 | 2.73 | 232.00 | 9.20 | 7 |
| 33c2 | 2 |  |  |  |  |  |  | 5 |
| 34c2 | 11036 | 21 | 6 | 5 | 0.19 | 36.00 | 1.06 | 12 |
| 35c2 | 13 |  |  |  |  |  |  | 8 |
| 36c2 | 5099 | 24 | 6 | 2 | 0.10 | 31.86 | 1.02 | 9 |
| 25d2 | 2953 | 33 | 22 | 14 | 1.70 | 60.50 | 2.78 | 21 |
| 26d2 | 548 | 13 | 13 | 11 | 2.06 | 13.00 | 5.68 | 13 |
| 27d2 | 466 | 22 |  | 14 | 1.76 | 25.33 | 3.17 | 13 |
| 31d2 | 852 | 34 | 27 | 15 | 1.33 | 43.75 | 1.99 |  |
| 32d2 | 763 | 29 | 27 | 15 | 2.60 | 31.50 | 9.72 | 8 |
| 33d2 | 533 | 25 | 24 | 18 | 2.34 | 34.33 | 7.26 | 9 |
| 34d2 | 1357 | 7 | 4 | 1 | 0.04 | 22.00 | 1.01 | 8 |
| 35d2 | 1253 | 52 | 40 | 26 | 2.71 | 74.67 | 8.63 | 9 |
| 36d2 | 923 | 6 | 3 | 1 | 0.06 | 7.00 | 1.02 | 10 |
| 25s10 | 279 | 26 |  | 17 | 2.11 | 41.60 | 5.89 |  |
| 26s10 | 91 | 17 |  |  | 2.28 | 20.75 | 7.13 | 18 |
| 27s10 | 5129 | 17 | 9 | 7 | 1.57 | 20.00 | 3.57 | 5 |
| 31s10 | 2795 | 32 | 22 | 12 | 1.81 | 35.00 | 3.57 | 13 |
| 32s10 | 12389 | 84 | 24 | 14 | 1.90 | 151.36 | 3.27 | 10 |
| 33s10 | 5427 | 55 | 31 | 17 | 2.33 | 118.33 | 6.05 | 8 |
| 34s10 | 9634 | 11 | 1 | 1 | 0.02 | 16.00 | 1.00 | 9 |
| 35s10 | 4621 | 101 | 43 | 28 | 2.74 | 138.71 | 6.67 | 9 |
| 36s10 | 13476 | 22 | 3 | 1 | 0.04 | 61.00 | 1.01 | 8 |
| 25c10 | 7031 | 67 | 29 | 12 | 1.78 | 85.07 | 2.59 | 15 |
| 26c10 | 5413 | 52 | 28 | 16 | 1.80 | 78.25 | 2.77 | 21 |
| 27c10 | 332 | 20 |  | 12 | 1.04 | 27.20 | 1.62 | 11 |
| 31c10 | 4744 | 61 | 32 | 15 | 2.07 | 88.14 | 4.51 | 2 |
| 32c10 | 310 | 26 |  | 17 | 2.61 | 31.00 | 9.56 | 8 |
| 33c10 | 1226 | 68 | 53 | 31 | 3.04 | 94.25 | 10.43 | 8 |
| 34c10 | 149 | 6 |  | 6 | 0.41 | 9.00 | 1.20 | 12 |
| 35c10 | 9585 | 120 | 44 | 22 | 2.81 | 191.75 | 8.72 | 11 |
| 36c10 | 237 | 5 |  | 3 | 0.25 | 6.00 | 1.11 | 10 |
| 25d10 | 2928 | 39 | 23 | 13 | 2.15 | 52.75 | 5.10 | 16 |
| 26d10 | 128 | 3 |  | 3 | 0.48 | 3.00 | 1.38 |  |
| 27d10 | 5176 | 29 | 16 | 11 | 1.84 | 31.14 | 4.22 | 13 |
| 31d10 | 110 | 6 |  | 6 | 0.76 | 7.00 | 1.53 | 8 |
| 32d10 | 8887 | 101 | 38 | 25 | 2.98 | 162.50 | 13.90 | 7 |
| 33d10 | 101 | 13 |  | 13 | 1.25 | 18.00 | 1.97 | 10 |
| 34d10 | 5846 | 4 | 1 | 1 | 0.00 | 7.00 | 1.00 | 10 |
| 35d10 | 251 | 13 |  | 9 | 1.37 | 14.00 | 2.42 | 8 |
| 36d10 | 4450 | 23 | 8 | 5 | 0.29 | 32.00 | 1.11 | 8 |
