# Supplementary material for: Patchiness of Ciliate Communities Sampled at Varying Spatial Scales along the New England Shelf
Source: PLoS One. 2016 Dec 9;11(12):e0167659. doi: 10.1371/journal.pone.0167659 (PMC5147948; doi:10.1371/journal.pone.0167659)
Supplement: S2 Table — (DOCX) [file pone.0167659.s008.docx]

**S2 Table:** OTU best BLAST results to a morphospecies.

| OTU | Occurrence | Reads | Morphospecies | | accession number | identity |
| --- | --- | --- | --- | --- | --- | --- |
| OTU329 | 60 | 59015 | *Pelagostrobilidium paraepacrum* | | FJ876963.1 | 95.21% |
| OTU2165 | 132 | 317530 | *Salpingella acuminata* | | EU399536.1 | 99.36% |
| OTU2594 | 124 | 103127 | *Strombidium cf. basimorphum* | | JF791016.1 | 100.00% |
| OTU2176 | 105 | 113108 | *Stenosemella pacifica* | | JN831794.1 | 100.00% |
| OTU3046 | 114 | 150656 | *Amphorides quadrilineata* | | JX101850.1 | 100.00% |
| OTU2678 | 66 | 72685 | *Salpingella acuminata* | | EU399536.1 | 96.79% |
| OTU2373 | 129 | 63964 | *Strombidium basimorphum* | | FJ480419.1 | 99.36% |
| OTU3011 | 128 | 134927 | *Strombidium* sp. | | AY143564.1 | 98.09% |
| OTU1970 | 99 | 12744 | *Pseudotontonia* sp. | | JX178820.1 | 99.04% |
| OTU2986 | 116 | 48200 | *Eutintinnus stramentus* | | JX101859.1 | 100.00% |
| OTU2644 | 104 | 60994 | ClusterX2 | |  | 92.78% |
| OTU2101 | 43 | 16497 |  |  |  |  |
| OTU560 | 64 | 7916 | *Heterocapsa triquetra* | | GU594638.1 | 100.00% |
| OTU2408 | 73 | 8920 | ClusterX2 | |  | 98.97% |
| OTU1955 | 86 | 16096 | *Pelagostrobilidium neptuni* | | AY541683.1 | 100.00% |
| OTU814 | 68 | 12189 | *Rimostrombidium veniliae* | | FJ876964.1 | 95.83% |
| OTU1368 | 37 | 19129 | *Salpingella acuminata* | | EU399536.1 | 98.40% |
| OTU877 | 54 | 5328 | ClusterX2 | |  | 92.44% |
| OTU2806 | 80 | 16570 | *Tintinnopsis lata* | | KM982810.1 | 100.00% |
| OTU1010 | 61 | 9848 | *Rimostrombidium veniliae* | | FJ876964.1 | 94.87% |
| OTU3045 | 54 | 27086 | *Salpingella acuminata* | | EU399536.1 | 94.23% |
| OTU2427 | 85 | 24764 | *Pseudotontonia simplicidens* | | JF791015.1 | 100.00% |
| OTU1484 | 15 | 18079 | *Salpingella acuminata* | | EU399536.1 | 97.12% |
| OTU526 | 78 | 4255 | *Salpingella acuminata* | | EU399536.1 | 99.04% |
| OTU1412 | 25 | 6423 | *Rimostrombidium veniliae* | | FJ876964.1 | 93.27% |
| OTU1413 | 24 | 2486 | *Eutintinnus fraknoi* | | EU399534.1 | 99.68% |
| OTU2124 | 8 | 4901 | *Salpingella acuminata* | | EU399536.1 | 97.76% |
| OTU72 | 42 | 981 | *Tintinnopsis* sp*.* | | JN831850.1 | 97.74% |
| OTU1530 | 38 | 10236 | *Salpingella acuminata* | | EU399536.1 | 98.08% |
| OTU2860 | 12 | 3627 | *Salpingella acuminata* | | EU399536.1 | 98.40% |
| OTU2301 | 47 | 1320 | *Salpingella acuminata* | | EU399536.1 | 96.49% |
| OTU136 | 11 | 4339 | *Rimostrombidium veniliae* | | FJ876964.1 | 94.55% |
| OTU3051 | 27 | 29727 | *Pelagostrobilidiumparaepacrum* | | FJ876963.1 | 96.50% |
| OTU288 | 20 | 8713 | ClusterX2 | |  | 93.13% |
| OTU2971 | 37 | 2095 | *Strombidium basimorphum* | | FJ480419.1 | 98.73% |
| OTU1472 | 54 | 12072 | *Undella marsupialis* | | JQ408214.1 | 100.00% |
| OTU2156 | 19 | 820 | *Dadayiella ganymedes* | | JX101853.1 | 97.10% |
| OTU1852 | 20 | 3556 | *Favella campanula* | | JX101861.1 | 94.84% |
| OTU1448 | 26 | 1078 | *Novistrombidium sinicum* | | FJ422990.1 | 97.44% |
| OTU1175 | 32 | 9739 | *Strombidinopsis* sp*.* | | AF399135.1 | 99.37% |
| OTU657 | 35 | 3285 | *Strombidium basimorphum* | | FJ480419.1 | 98.73% |
| OTU562 | 13 | 396 | ClusterX2 | |  | 91.75% |
| OTU624 | 21 | 358 | *Varistrombidium kielum* | | DQ811090.1 | 96.81% |
| OTU9 | 51 | 4234 | *Rhabdonella hebe* | | AY143566.1 | 100.00% |
| OTU1539 | 31 | 12996 | *Strombidium basimorphum* | | FJ480419.1 | 94.60% |
| OTU737 | 56 | 4456 | *Dadayiella ganymedes* | | JX101853.1 | 100.00% |
| OTU2786 | 2 | 162 | *Salpingella acuminata* | | EU399536.1 | 92.65% |
| OTU2532 | 30 | 3263 | *Pelagostrobilidium neptuni* | | AY541683.1 | 94.23% |
| OTU2334 | 14 | 517 | *Pelagostrobilidium neptuni* | | AY541683.1 | 94.23% |
| OTU487 | 18 | 1359 | *Lynnella semiglobulosa* | | FJ876965.1 | 97.72% |
| OTU1670 | 36 | 1208 | *Strombidium basimorphum* | | FJ480419.1 | 97.77% |
| OTU45 | 55 | 1513 | *Salpingella acuminata* | | EU399536.1 | 98.08% |
| OTU3077 | 8 | 190 | *Pelagostrobilidium minutum* | | FJ876959.1 | 99.04% |
| OTU2609 | 1 | 210 | *Eutintinnus pectinis* | | AF399170.1 | 100.00% |
