# Supplementary material for: Patchiness of Ciliate Communities Sampled at Varying Spatial Scales along the New England Shelf
Source: PLoS One. 2016 Dec 9;11(12):e0167659. doi: 10.1371/journal.pone.0167659 (PMC5147948; doi:10.1371/journal.pone.0167659)
Supplement: S3 Table — Some abundant OTUs are related to the distance to the shore and to the depth (e.g. OTU329 mainly observed inshore or OTU2594 mainly observed within the surface layers in the offshore location) as observed in Fig 4. (DOCX) [file pone.0167659.s009.docx]

**S3 Table:** Pearson correlations show complex relationship between OTUs and environmental parameters. Some abundant OTUs are related to the distance to the shore and to the depth (e.g. OTU329 mainly observed inshore or OTU2594 mainly observed within the surface layers in the offshore location) as observed in Figure 4.
